# Scrotal Migration of the Ventriculoperitoneal Shunt in a 1-Year-Old Pediatric Patient: A Case Report and Systematic Literature Review

**DOI:** 10.3390/jcm14155183

**Published:** 2025-07-22

**Authors:** Zenon Pogorelić, Stipe Ninčević, Vlade Babić, Miro Jukić, Stipe Vidović

**Affiliations:** 1 Department of Pediatric Surgery, University Hospital of Split, 21000 Split, Croatia; 2 Department of Surgery, School of Medicine, University of Split, 21000 Split, Croatia; 3 Department of Neurosurgery, University Hospital of Split, 21000 Split, Croatia; 4 Faculty of Medicine Osijek, University of Osijek, 31000 Osijek, Croatia

**Keywords:** ventriculoperitoneal shunt, shunt migration, scrotal migration, hydrocephalus, processus vaginalis, scrotal swelling, children, complication, laparoscopy

## Abstract

**Background:** Migration of the peritoneal end of the ventriculoperitoneal shunt (VPS) into the scrotum is a rare but recognized complication. Inguinoscrotal migration typically occurs as a result of increased intra-abdominal pressure combined with a patent processus vaginalis. A 14-month-old pediatric patient presented to the emergency department with abdominal pain, vomiting, and swelling of the right scrotum that had persisted for two days. The patient had a history of a head injury that had resulted in a large secondary arachnoid cyst for which a VPS had been placed at eight months of age. Examination of the inguinoscrotal region revealed a swollen and painful right side of the scrotum with a hydrocele and a palpable distal portion of the ventriculoperitoneal catheter in the right groin extending to the scrotum. After a brief preoperative preparation, the patient underwent laparoscopic abdominal emergency exploration, during which shunt repositioning and laparoscopic closure of the patent processus vaginalis were performed. **Methods**: A systematic review was conducted in accordance with the Preferred Reporting Items for Systematic Reviews and Meta-Analyses (PRISMA) guidelines. **Results:** A total of 30 case reports and six case series were included, analyzing 52 pediatric patients with scrotal migration of the VPS. The median age at presentation was 24 months (range: 1–169 months). The indication for VPS placement was hydrocephalus. Migration of the VPS catheter occurred on the right side in 34 cases. The median interval from VPS placement to the onset of symptoms was 9.0 months (range: 1 day–72 months). The most frequently reported symptoms were scrotal/inguinoscrotal swelling (*n* = 50), vomiting (*n* = 7), and fever (*n* = 3). Diagnostic methods included abdominal X-ray (*n* = 43), ultrasound (*n* = 5), scrotal transillumination test (*n* = 5), and computed tomography (*n* = 1). Regarding treatment, surgical repositioning of the VPS catheter into the peritoneal cavity was performed in 47 patients (90.4%), with no intraoperative or postoperative complications reported. **Conclusions:** Laparoscopic repositioning of the VPS into the peritoneal cavity, combined with closure of the processus vaginalis, appears to be a safe and effective treatment option for scrotal migration of the VPS. However, further well-designed studies are warranted to provide more comprehensive, generalizable, and unbiased evidence regarding this complication in the pediatric population.

## 1. Introduction

Hydrocephalus is defined as the accumulation of cerebrospinal fluid (CSF) within the cerebral ventricles, resulting from either primary (congenital, developmental, or genetic) or secondary causes, such as central nervous system (CNS) infections, meningitis, brain tumors, head trauma, or spontaneous intracranial hemorrhage [1]. Based on the underlying mechanism, hydrocephalus can be classified into communicating, non-communicating (obstructive), ex-vacuo hydrocephalus, and normal pressure hydrocephalus [2].

The ventriculoperitoneal shunt (VPS) remains the most commonly performed surgical treatment for hydrocephalus in the pediatric population and is currently the most frequent procedure in pediatric neurosurgery [3,4]. A typical VPS system consists of two components: a proximal catheter that diverts CSF from the ventricles, and a distal catheter that typically terminates in the peritoneal cavity. Other alternative distal sites include the right atrium (ventriculoatrial shunt), pleural space (ventriculopleural shunt), and the lumbar subarachnoid space connected to the peritoneum (lumboperitoneal shunt) [5]. It is estimated that approximately 30,000 shunt procedures are performed annually in the United States. VPS-related complications may be classified as mechanical, infectious, or functional [6]. Functional complications include CSF overdrainage, valve malfunction, catheter breakage, obstruction, coiling, or spontaneous knot formation [7,8].

The most common causes of shunt failure in both children and adults are obstruction and infection, with infection typically leading to early shunt failure, while catheter obstruction is more often associated with late failure [8,9]. Studies have identified several risk factors for shunt malfunction, including patient age, prior surgeries before shunt placement, the underlying etiology of hydrocephalus, and the specific type of hydrocephalus [10]. Patients with congenital hydrocephalus or spinal dysraphism have been shown to have a significantly higher incidence of shunt malfunction compared to those with other etiologies. In contrast, patients with normal pressure hydrocephalus (NPH) exhibit the lowest rates of shunt revision [11].

Mechanical complications of the VPS include migration of the catheter into the thoracic cavity, heart, bladder, hernia sacs, anus, and distal part of the scrotum, which can lead to infection and/or inadequate CSF drainage, which, in turn, can cause obstructive hydrocephalus [12]. Although cases of scrotal migration of the distal catheter have been reported in adults, they are rare due to obliteration of the processus vaginalis [7]. Consequently, this complication is more commonly observed in pediatric patients, where the processus vaginalis often remains patent. Scrotal migration may lead to more serious clinical manifestations, including scrotal edema, acute scrotum, abdominal pain, and even shunt extrusion [13].

In the past, the open surgical approach was considered the gold standard for the treatment of inguinal hernia or a patent processus vaginalis. The most commonly employed technique involved high ligation of the hernia sac [14]. However, with significant advancements in minimally invasive pediatric surgery, instruments have been developed that enable these procedures to be performed even in neonates and small children. As a result, virtually all pediatric abdominal surgeries can now be carried out laparoscopically [15]. In recent years, the percutaneous internal ring suturing (PIRS) technique has gained particular popularity among pediatric surgeons for the treatment of indirect inguinal hernias, offering excellent outcomes and very low recurrence rates [16].

In this report, we present a rare case of a VPS catheter loop within the right scrotum of a 14-month-old pediatric patient, which resulted in a painful hydrocele and was successfully managed using a laparoscopic approach. In addition, we present the findings of a systematic review of the literature.

## 2. Case Presentation

A 14-month-old pediatric patient presented to the emergency department of our hospital with abdominal pain, vomiting, and swelling of the right scrotum that had persisted for two days. The patient had a history of head injury resulting in a large secondary arachnoid cyst, for which a VPS had been placed at 8 months of age. Until two days before admission, the patient was in good general condition, growing and developing normally, and the VPS was functioning normally.

On physical examination, the patient was anxious and irritable, but hemodynamically stable and afebrile. The abdomen was soft, with no signs of peritoneal guarding. Examination of the inguinoscrotal region revealed a swollen and painful right side of the scrotum with a hydrocele and a palpable distal portion of the VP (ventriculoperitoneal) catheter in the right groin extending towards the scrotum. The scrotal ultrasound revealed a normal testicle, with no signs of inflammation, swelling, or torsion.

**Figure 1:**
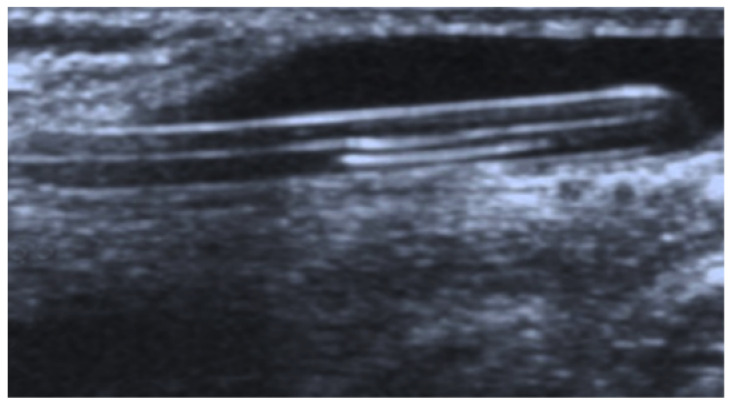
Ultrasound of the scrotum—a moderate hydrocele and tubular echogenic material corresponding to the distal end of the VP catheter.

A scrotal ultrasound was performed, which showed a moderate hydrocele on the right side and the individualization of tubular echogenic material therein, which was consistent with the distal end of the VP catheter (Figure 1).

**Figure 2:**
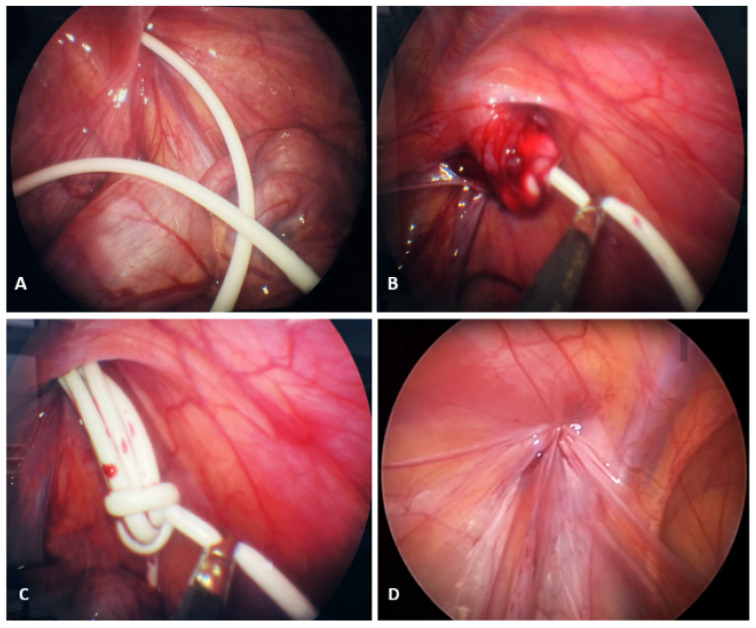
Intraoperative findings: (**A**)—VP catheter in the abdomen, the end of which has entered the inguinal canal and scrotum through an open processus vaginalis; (**B**)—Attempt to reposition the VP catheter in the abdominal cavity; (**C**)—After complete withdrawal of the catheter, the long part of the catheter was found to be in the scrotum and buried around its axis; (**D**)—Closure of the internal ring using the PIRS method.

After a brief preoperative preparation, the patient underwent an emergency laparoscopic abdominal exploration. A supraumbilical incision was made, a 5 mm trocar was inserted, and an 8-mmHg pneumoperitoneum was established. Exploration of the abdominal cavity revealed a VP catheter in the abdomen, the end of which entered the inguinal canal and scrotum through an open processus vaginalis (Figure 2A). After the insertion of two additional lateral 3.5 mm trocars, an attempt was made to reposition the VP catheter in the abdominal cavity using gentle movements, but this was met with resistance. Even after stronger traction, it was not possible to pull the catheter into the abdominal cavity (Figure 2B), which is why the laparoscopic incision of the hernia sac was performed using laparoscopic scissors, and the catheter was pulled out of the scrotum into the abdominal cavity, after the adhesions had been dissected. After the catheter had been pulled out completely, it was found that the long part of the catheter was lying in the scrotum and was buried around its axis (Figure 2C). The catheter was repositioned in the pelvis. The internal opening of the inguinal canal was then closed using the percutaneous internal ring suturing (PIRS) method [17], as shown in Figure 2D. At the end of the procedure, the VP catheter was checked and found to be functioning normally. The skin incisions were closed with Steri-Strip adhesive bands (3M^TM^ Steri-StripTM, Neuss, Germany).

After surgery, the patient was observed in the pediatric surgery department. Oral intake was initiated two hours after surgery. Ibuprofen at a dose of 10 mg/kg was used for analgesia. The patient was discharged from the hospital after 24 h in good general condition, pain-free, and afebrile. At the follow-up examination seven days later, the Steri-Strip adhesive bands were removed, and the surgical incisions had healed well. At six-month follow-up, the patient is in good general condition, pain-free, the VP catheter is functioning properly, and there are no signs of recurrent inguinal hernia.

## 3. Methods

The following paragraphs relate to the methodology of the systematic review on scrotal migration of the VPS in the pediatric population.

### 3.1. Inclusion and Exclusion Criteria

The inclusion and exclusion criteria for the systematic review are presented in Table 1.

### 3.2. Data Sources and Search Strategy

A systematic review was conducted in accordance with the Preferred Reporting Items for Systematic Reviews and Meta-Analyses (PRISMA) guidelines. A literature search was performed by reviewers S.V. and Z.P. on 10 April 2025, across four electronic databases: PubMed, ScienceDirect, Scopus, and Web of Science. Boolean logic expressions were used for the search without applying any filters, as follows: PubMed: ((ventriculoperitoneal shunt) AND (scrotal migration)); Scopus: ((ventriculoperitoneal shunt) AND (scrotal migration)); ScienceDirect: ((ventriculoperitoneal shunt) AND (scrotal migration)); and Web of Science: TS = ((ventriculoperitoneal shunt) AND (scrotal migration)). In addition to the electronic search, a manual screening of the reference lists from the selected articles was conducted by reviewers S.V. and Z.P. to identify any further relevant studies.

### 3.3. Study Selection and Data Collection Process

Following the removal of duplicate records, reviewers S.V. and Z.P. collaboratively screened the titles and abstracts of all articles retrieved through the electronic database search. Studies selected for full-text review were identified according to the predefined inclusion and exclusion criteria (Table 1). After evaluating the full texts, articles that did not meet the eligibility criteria were excluded, with reasons for exclusion recorded. Additionally, S.V. and Z.P. manually reviewed the reference lists of the included studies to identify and incorporate any further eligible articles.

For each study included in the systematic review, data extraction was carried out by reviewers S.V. and Z.P. The following information was collected when available: author(s), year of publication, country of origin, study design, and sample size. Patient-related data were also extracted, including age, indication for VPS placement, presenting symptoms, time to clinical presentation following VPS insertion, imaging and laboratory findings, presence of inguinal hernia, type of treatment administered, intraoperative and postoperative complications, length of hospital stay, mortality, and duration of follow-up for patients with scrotal migration of the VPS.

### 3.4. Risk of Bias Assessment of Included Studies

To evaluate the methodological quality and potential bias of the studies included in the review, the Joanna Briggs Institute (JBI) Critical Appraisal Checklist for Case Reports and the JBI Critical Appraisal Checklist for Case Series were utilized [18]. Two reviewers (S.V. and Z.P.) independently assessed each item on the relevant checklist, assigning one of four possible responses: ‘Yes’, ‘No’, ‘Unclear’, or ‘Not applicable’. Discrepancies between the reviewers were addressed and resolved through discussion. For scoring, each ‘Yes’ response was awarded one point, while ‘No’, ‘Unclear’, and ‘Not applicable’ responses received zero points. The total score was determined by summing the points from all ‘Yes’ responses and was then converted into a percentage by dividing by the maximum possible score. Based on this percentage, the methodological quality of each study was classified as low (<50%), moderate (50–74%), or high (>75%).

### 3.5. Statistical Analysis

Statistical analysis was performed using the Statistical Package for Social Sciences (SPSS, Version 28.0; IBM Corp., Armonk, NY, USA). The normality of numerical data was assessed using the Shapiro–Wilk test. As the distributions were non-normal, numerical variables were expressed as medians and interquartile ranges (IQRs), while categorical variables were summarized using absolute numbers and relative frequencies (percentages). Missing data were not imputed; analyses were based on available data only. For each variable analyzed, percentages were calculated using only the total number of participants from studies that reported data for that specific variable. Studies that did not provide data for a given variable were excluded from the denominator in those calculations. 

## 4. Results

### 4.1. Study Selection

**Figure 3:**
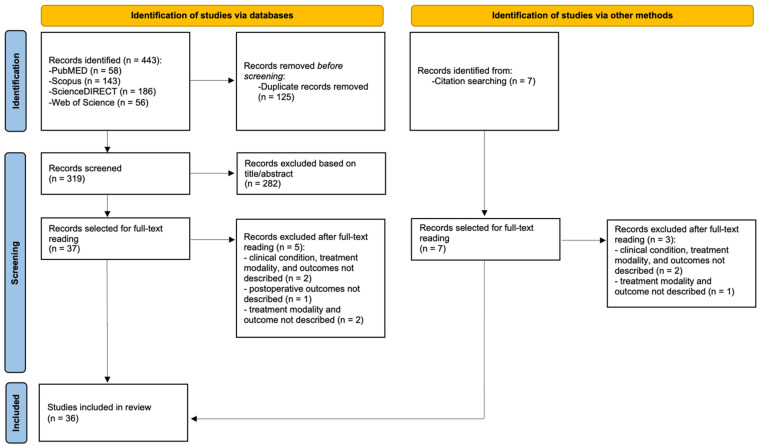
PRISMA flow diagram.

The database search initially identified 443 studies, of which 125 were duplicates. Based on the screening of titles and abstracts according to the predefined inclusion and exclusion criteria (Table 1), 282 records were excluded. Subsequently, 37 articles were selected for full-text review, after which 5 were excluded due to the absence of a described clinical condition, treatment modality, or reported outcomes. In addition, a manual search of reference lists identified seven more studies, of which three were excluded after full-text assessment for the same reasons. Ultimately, 36 studies were included in the systematic review. The literature search flow diagram is presented in Figure 3.

### 4.2. Study Characteristics

Ultimately, the review incorporated 30 case reports and six case series, and the key characteristics of these studies are presented in Table 2.

### 4.3. Risk of Bias in Studies

Upon assessing the methodological quality and risk of bias using the JBI Critical Appraisal Checklist for Case Series (Table 3) and the JBI Critical Appraisal Checklist for Case Reports (Table 4), 18 studies were classified as high quality, 16 as medium quality, and 2 as low quality based on the overall quality assessment score.

### 4.4. Summary of the Included Studies

A review and analysis of the 36 studies included in this article identified a total of 52 pediatric patients with VPS scrotal migration. The median age was 24 months (range: 1–169 months). For 51 patients, the indication for VPS placement was described, and all of them had hydrocephalus, of which 11 (21.6%) cases were congenital. Furthermore, the hydrocephalus was secondary to various underlying conditions, including myelomeningocele (*n* = 5, 9.8%), meningitis (*n* = 3, 5.9%), aqueductal stenosis (*n* = 3, 5.9%), tumor (*n* = 3, 5.9%), hemorrhage (*n* = 3, 5.9%), and Chiari malformation (*n* = 3, 5.9%). For 49 patients, the side of VPS migration was reported; of these, 34 (69.4%) occurred on the right side and 15 (30.6%) on the left side. The time from VPS placement to the onset of symptoms was available for 47 patients, with a median of 9.0 months (range: 1 day–72 months). The most commonly reported symptoms were scrotal/inguinoscrotal swelling (*n* = 50, 96.2%), vomiting (*n* = 7, 13.5%), and fever (*n* = 3, 5.8%). Diagnostic methods were reported for 49 patients and included abdominal X-ray (*n* = 43, 87.8%), ultrasound (*n* = 5, 10.2%), scrotal transillumination test (*n* = 5, 10.2%), and computed tomography (CT) (*n* = 1, 2.0%). An inguinal hernia was reported in 19 patients. Key characteristics and clinical findings of patients with VPS scrotal migration are summarized in Table 5.

Regarding treatment, surgical repositioning of the VPS into the peritoneal cavity was performed in 47 patients (90.4%). A laparoscopic approach was described in two patients (3.8%). Non-operative manual repositioning was reported in two cases (3.8%), and spontaneous resolution of VPS migration from the scrotum occurred in three patients (5.8%). The length of the hospital stay was reported in 15 patients, with a median duration of 3.2 days (range: 1–10 days). No intraoperative or postoperative complications were observed. Follow-up data were available for 12 patients, with a median follow-up period of 19.1 months (range: 1–120 months). Treatment approaches, intraoperative and postoperative complications, length of hospital stay, and mortality outcomes for patients with VPS scrotal migration are summarized in Table 6.

## 5. Discussion

This paper presents a relatively rare case of scrotal migration of the peritoneal end of a VPS in a one-year-old child. To our knowledge, it also represents the first systematic review of the literature focusing on scrotal migration of VPS in the pediatric population.

Several theories have been proposed to explain the scrotal migration of ventriculoperitoneal shunts (VPS), with the most widely accepted attributing it to increased intra-abdominal pressure [10,19]. This pressure can impede the natural obliteration of the processus vaginalis (PV), providing a potential pathway for distal catheter migration into the scrotum. This condition is especially prevalent in neonates and young children, in whom the PV remains open in up to 90% of cases at birth and gradually closes with age [52]. Furthermore, the combination of a smaller peritoneal cavity, the vertical orientation of the inguinal canal in early life, and the “funnel effect” created by a patent PV further facilitates this type of migration [30]. Collectively, these anatomical and physiological factors contribute to the increased risk of scrotal migration of the distal end of the VPS.

Studies indicate that the primary indication for VPS placement in children is hydrocephalus, which is often secondary to a variety of underlying conditions, including myelomeningocele, aqueductal stenosis, meningitis, brain tumors, intraventricular hemorrhage, and Chiari malformation [3,10,13,19,20,21]. In our case, the patient had a history of head trauma that led to the development of a large secondary arachnoid cyst, which necessitated VPS placement.

Findings from previous studies suggest that scrotal migration of VPS may occur more frequently on the right side, as was also observed in our case [3,10,13,19,20,21]. This pattern can be attributed to several anatomical and developmental factors. The processus vaginalis is known to remain patent longer on the right, increasing the likelihood of it serving as a pathway for shunt migration [52]. Additionally, right-sided inguinal hernias may be more common in the pediatric population, likely due to asymmetries in testicular descent and differences in the timing of processus vaginalis closure between the two sides [53,54,55]. The anatomical configuration of the peritoneal cavity may further contribute, as the spleen on the left side can act as a physical barrier, limiting catheter mobility in that direction [51]. Collectively, these factors may explain the observed predominance of right-sided VPS migration in children.

Furthermore, studies indicate that the most commonly reported symptoms associated with scrotal migration of VPS are scrotal swelling, vomiting, and fever (Table 5), which aligns with the clinical presentation observed in our patient. In addition to physical examination, ultrasound was used in our case to establish the diagnosis. According to the literature, the most frequently employed confirmatory diagnostic modality is abdominal X-ray, followed by ultrasound of the inguinoscrotal region, a positive transillumination test, and computed tomography (Table 4).

Regarding the treatment of VPS scrotal migration, studies indicate that the most common approach is surgical repositioning of the catheter into the peritoneal cavity (Table 6). In cases where an inguinal hernia is present, hernia repair is typically performed concurrently, as was performed in our case. No intraoperative or postoperative complications have been reported in the reviewed literature (Table 6). In our case, a laparoscopic approach was used, and to the best of our knowledge, this approach has been previously described only by Ezzat et al., who reported successful laparoscopic repositioning of the VPS into the peritoneal cavity in two patients, also without complications [18]. Given that only one other study, in addition to ours, has described a laparoscopic approach, a comparison with the open surgical technique is not feasible. Additionally, two studies reported manual (non-operative) repositioning of the catheter [23,40], while spontaneous resolution of scrotal migration was documented in three patients [3,37,49]. Additionally, two studies reported manual (non-operative) repositioning of the catheter [23,40], while spontaneous resolution of scrotal migration was documented in three patients [3,37,49]. The precise mechanism underlying the spontaneous resolution of scrotal migration remains unclear; however, it has been hypothesized that factors such as gravitational repositioning, fluctuations in intra-abdominal pressure, and progressive fibrotic encapsulation may contribute to the gradual return of the distal catheter to its intended anatomical position [3]. These findings suggest that conservative management may be a viable option in carefully selected asymptomatic patients, particularly within the pediatric population, where increased tissue elasticity and ongoing anatomical development may promote spontaneous correction [56]. In particular, conservative management might be justified in cases without clinical signs of shunt malfunction, infection, or hydrocele formation, and where the catheter remains functionally positioned despite scrotal descent. The absence of progressive symptoms and stable neuroimaging findings during follow-up may further support a watchful waiting approach [57]. Nonetheless, such an approach would require vigilant clinical monitoring to ensure early detection of potential shunt dysfunction or other complications. Given the rarity of these occurrences, further studies are warranted to elucidate predictive factors for spontaneous regression and to establish evidence-based guidelines for non-operative management [23].

### Limitations

One of the main limitations of this review is the relatively small number of included studies, the majority of which were case reports or single-center case series with limited sample sizes. This restricted the depth of analysis, introduced potential sources of bias, and significantly limited the generalizability of the findings. A meta-analysis was not performed, thereby reducing the ability to conduct a quantitative synthesis. As a result, interpretation of the findings relied more heavily on subjective judgment, the identification of consistent patterns across studies was hindered, and assessment of between-study heterogeneity was not feasible.

In addition, one case report [25] and one case series [24] were assessed as having low methodological quality according to the JBI Critical Appraisal Tools. Although these studies contributed relevant clinical observations, their methodological limitations may have introduced additional bias.

To address these limitations, future research should prioritize the design and implementation of high-quality retrospective cohort studies, randomized controlled trials, and prospective observational studies. Ideally, such studies should be conducted across multiple centers to improve the representativeness and robustness of the data. Furthermore, the adoption of standardized research protocols and consistent reporting of key clinical and demographic variables would facilitate more accurate cross-study comparisons and enable future meta-analyses.

## 6. Conclusions

This case report presents laparoscopic repositioning of the VPS into the peritoneal cavity, combined with closure of the processus vaginalis, as a safe and effective treatment strategy for managing scrotal migration of the VPS in pediatric patients. Further well-designed, multicenter studies are necessary to provide more comprehensive, generalizable, and unbiased evidence regarding this rare but clinically significant complication.

## Figures and Tables

**Table 1:** Inclusion and exclusion criteria.

	Inclusion Criteria	Exclusion Criteria
Period of the study	All available literature to date	/
Language	English	Languages that are not English
Study design	Case report, retrospective study (case–control studies or case series), meta-analysis, and systematic review	Conference abstracts, commentaries, personal communications, discussion, and editorials
Participants	<18 years	>18 years
Study topic	Ventriculoperitoneal shunt with scrotal migration	Ventriculoperitoneal shunt with non-scrotal migration

**Table 2:** Key characteristics of included studies.

Author	Year of Publication	Country	Study Design	Sample Size
Muhajir et al. [19]	2025	Indonesia	Case series	3
Javeed et al. [10]	2024	Pakistan	Case report	1
Topp et al. [20]	2023	Albany	Case report	1
Chanchlani et al. [21]	2023	India	Case report	1
Taha et al. [13]	2022	Egypt	Case report	1
Alkhudari et al. [3]	2022	Saudi Arabia	Case report	1
Ahmed et al. [22]	2021	Yemen	Case report	1
Hauser et al. [23]	2020	Austria	Case report	1
Abdoli et al. [24]	2019	Iran	Case series	4
Agarwal et al. [25]	2019	India	Case report	1
Dharmajaya [26]	2018	Indonesia	Case report	1
Ezzat et al. [27]	2018	Germany	Case series	3
Paterson et al. [28]	2018	Australia	Case report	1
Nawaz et al. [29]	2018	Pakistan	Case report	1
Bawa et al. [30]	2017	India	Case series	4
Hung et al. [31]	2017	Taiwan	Case report	1
Ricci et al. [32]	2016	USA	Case report	1
Shankar et al. [33]	2014	India	Case report	1
Erikci et al. [34]	2013	Turkey	Case report	1
Panda et al. [35]	2013	India	Case report	1
Shahizon et al. [36]	2013	Malaysia	Case report	1
Ramareddy et al. [37]	2012	India	Case report	1
Gupta et al. [38]	2012	India	Case report	1
Mohammadi et al. [39]	2012	Iran	Case report	1
Kita et al. [40]	2010	Japan	Case report	1
Rahman et al. [41]	2009	UK	Case report	1
Ward et al. [42]	2001	Japan	Case report	1
Öktem et al. [43]	1998	Turkey	Case series	4
Ammar et al. [44]	1991	Saudi Arabia	Case report	1
Calvario et al. [45]	1991	Brazil	Case report	1
Kwok et al. [46]	1989	Hong Kong	Case report	1
Ram et al. [47]	1987	Israel	Case report	1
Fuwa et al. [48]	1984	Japan	Case report	1
Crofford et al. [49]	1983	USA	Case series	4
Bristow et al. [50]	1978	USA	Case report	1
Levey et al. [51]	1977	USA	Case report	1

**Table 3:** Methodological quality of included study according to JBI Critical Appraisal Checklist for Case Series.

	Methodological Quality of Included Study According to JBI Critical Appraisal Checklist for Case Report										
	Q1	Q2	Q3	Q4	Q5	Q6	Q7	Q8	Q9	Q10	Overall Score
Muhajir et al. [19]	Yes	Yes	Yes	U	Yes	Yes	Yes	No	Yes	NA	70% (Medium quality)
Abdoli et al. [24]	Yes	No	Yes	U	Yes	No	No	No	Yes	NA	40% (Low quality)
Ezzat et al. [27]	Yes	Yes	Yes	Yes	Yes	Yes	No	No	Yes	NA	70% (Medium quality)
Bawa et al. [30]	Yes	Yes	Yes	Yes	Yes	Yes	Yes	No	No	Yes	80% (High quality)
Öktem et al. [43]	Yes	Yes	Yes	Yes	Yes	Yes	Yes	No	No	NA	70% (Medium quality)
Crofford et al. [49]	Yes	Yes	Yes	Yes	Yes	Yes	No	No	Yes	NA	70% (Medium quality)

Q1 = Were there clear criteria for inclusion in the case series?, Q2 = Was the condition measured in a standard, reliable way for all participants included in the case series?, Q3 = Were valid methods used for identification of the condition for all participants included in the case series?, Q4 = Did the case series have consecutive inclusion of participants?, Q5 = Did the case series have complete inclusion of participants?, Q6 = Was there clear reporting of the demographics of the participants in the study?, Q7 = Was there clear reporting of clinical information of the participants?, Q8 = Were the outcomes or follow-up results of cases clearly reported?, Q9 = Was there clear reporting of the presenting site(s)/clinic(s) demographic information?, Q10 = Was statistical analysis appropriate?, U = Unclear, NA = Not applicable.

**Table 4:** Methodological quality of the included study according to the JBI Critical Appraisal Checklist for Case Report.

	Methodological Quality of the Included Study According to the JBI Critical Appraisal Checklist for Case Report								
	Q1	Q2	Q3	Q4	Q5	Q6	Q7	Q8	Overall Score
Javeed et al. [10]	Yes	Yes	Yes	Yes	Yes	Yes	Yes	Yes	100% (High quality)
Topp et al. [20]	Yes	Yes	Yes	Yes	Yes	Yes	Yes	Yes	100% (High quality)
Chanchlani et al. [21]	Yes	Yes	Yes	Yes	No	No	No	Yes	62.5% (Medium quality)
Taha et al. [13]	Yes	Yes	Yes	Yes	Yes	Yes	Yes	Yes	100% (High quality)
Alkhudari et al. [3]	Yes	Yes	Yes	Yes	Yes	Yes	Yes	Yes	100% (High quality)
Ahmed et al. [22]	Yes	Yes	Yes	No	No	No	Yes	Yes	62.5% (Medium quality)
Hauser et al. [23]	Yes	Yes	Yes	Yes	Yes	No	No	Yes	75% (Medium quality)
Agarwal et al. [25]	Yes	No	No	Yes	Yes	No	Yes	No	50% (Low quality)
Dharmajaya [26]	Yes	Yes	Yes	Yes	Yes	Yes	No	Yes	87.5% (High quality)
Paterson et al. [28]	Yes	Yes	Yes	Yes	Yes	No	Yes	Yes	87.5% (Medium quality)
Nawaz et al. [29]	Yes	Yes	Yes	Yes	Yes	Yes	Yes	Yes	100% (High quality)
Hung et al. [31]	Yes	Yes	Yes	Yes	Yes	No	Yes	Yes	87.5% (High quality)
Ricci et al. [32]	Yes	Yes	Yes	Yes	Yes	No	Yes	Yes	87.5% (High quality)
Shankar et al. [33]	Yes	Yes	Yes	Yes	Yes	No	No	Yes	75% (Medium quality)
Erikci et al. [34]	Yes	Yes	Yes	Yes	Yes	Yes	Yes	Yes	100% (High quality)
Panda et al. [35]	Yes	Yes	Yes	Yes	Yes	Yes	Yes	Yes	100% (High quality)
Shahizon et al. [36]	Yes	Yes	Yes	Yes	Yes	No	Yes	Yes	87.5% (High quality)
Ramareddy et al. [37]	Yes	Yes	Yes	Yes	Yes	Yes	Yes	Yes	100% (High quality)
Gupta et al. [38]	Yes	Yes	Yes	Yes	Yes	No	No	Yes	75% (Medium quality)
Mohammadi et al. [39]	Yes	Yes	Yes	Yes	Yes	No	Yes	Yes	87.5% (High quality)
Kita et al. [40]	Yes	Yes	Yes	Yes	Yes	No	No	No	62.5% (Medium quality)
Rahman et al. [41]	Yes	Yes	Yes	Yes	Yes	No	No	Yes	75% (Medium quality)
Ward et al. [42]	Yes	Yes	Yes	Yes	Yes	No	Yes	Yes	87.5% (High quality)
Ammar et al. [44]	Yes	Yes	Yes	Yes	Yes	No	Yes	Yes	87.5% (High quality)
Calvario et al. [45]	Yes	Yes	Yes	Yes	No	No	No	Yes	62.5% (Medium quality)
Kwok et al. [46]	Yes	Yes	Yes	Yes	Yes	No	No	Yes	75% (Medium quality)
Ram et al. [47]	Yes	Yes	Yes	Yes	Yes	No	No	Yes	75% (Medium quality)
Fuwa et al. [48]	Yes	Yes	Yes	Yes	Yes	No	NA	Yes	75% (Medium quality)
Bristow et al. [50]	Yes	Yes	Yes	Yes	Yes	No	Yes	Yes	87.5% (High quality)
Levey et al. [51]	Yes	Yes	Yes	Yes	Yes	No	Yes	Yes	87.5% (High quality)

Q1 = Were the patient’s demographic characteristics clearly described?, Q2 = Was the patient’s history clearly described and presented as a timeline?, Q3 = Was the current clinical condition of the patient on presentation clearly described?, Q4 = Were diagnostic tests or assessment methods and the results clearly described?, Q5 = Was the intervention(s) or treatment procedure(s) clearly described?, Q6 = Was the post-intervention clinical condition clearly described?, Q7 = Were adverse events (harms) or unanticipated events identified and described?, Q8 = Does the case report provide takeaway lessons? NA = Not applicable.

**Table 5:** Key characteristics and clinical findings of patients with VPS scrotal migration.

	Age (Months)	Indications for VPS	Side	Time to Clinical Presentation After Shunting	Symptoms	Imaging and Laboratory Findings	Inguinal Hernia
Muhajir et al. [19]	5, 10, 6	Case 1: Congenital hydrocephalus Case 2: Hydrocephalus secondary to aqueductal stenosis Case 3. Multiloculated hydrocephalus Dandy–Walker variant	L, R, R	4, 11, and 4 months	Case 1: Vomiting, palpable distal tip catheter in the scrotum Case 2: Seizures, testicular swelling Case 3: Vomiting, enlarged abdomen with a lump, and redness on the scrotum	Cases 1.–3.: Abdominal X-ray	No
Javeed et al. [10]	7	Hydrocephalus due to myelomeningocele	R	13 days	Five-day history of scrotal swelling, vomiting, and irritability.	Abdominal X-ray	No
Topp et al. [20]	16	Hydrocephalus due to myelomeningocele and Chiari II malformation	R	15 months	Two-day history of scrotal swelling, clear fluid draining from the scrotal sac, emesis, and progressive lethargy.	US, CT Leukocytosis and anion gap acidosis.	R
Chanchlani et al. [21]	2	Communicating hydrocephalus	R	5 days	Scrotal swelling	Abdomen X-ray	R
Taha et al. [13]	3	Hydrocephalus due to myelomeningocele	R	NA	Seven-day history of scrotal swelling.	US, abdominal X-ray; Positive translumination	No
Alkhudari et al. [3]	6	Hydrocephalus secondary to intraventricular hemorrhage (gradus III)	R	NA	Right inguinoscrotal swelling, and a 15-day history of vomiting after each feed, constipation for 6 days	Abdominal X-ray	No
Ahmed et al. [22]	8	Hydrocephalus	L	7 months	Seven-day history of left scrotal swelling and fever.	Abdomen X-ray, Positive transillumination	L
Hauser et al. [23]	23	Hydrocephalus due to closed myelomeningocele accompanied by septum pellucidum agenesis, corpus callosum hypoplasia, and Chiari type II malformation	R	NA	2-day history of painless scrotal swelling	Physical examination (palpable tube inside the scrotum).	R
Abdoli et al. [24]	24, 12, 18, 12	Case 1: Hydrocephalus Case 2: Hydrocephalus Case 3: Congenital hydrocephalus Case 4: Hydrocephalus	R, R, R, L	10 days, 10 months, 5 months, and 8 months	Case 1. Right scrotal swelling Case 2: Inguinal herniation Case 3. Right inguinal region swelling Case 4. Inguinoscrotal swelling	Case 1. Abdominal X-ray Case 2: Intraoperative findings Case 3–4. Surgical exploration	R, R, R, L
Agarwal et al. [25]	14	NA	R	7 months	Right scrotal swelling	Abdominal X-ray	No
Dharmajaya [26]	12	Communicating hydrocephalus	R	7 months	Right scrotal swelling (slowly grown over 3 days)	Abdominal X-ray	No
Ezzat et al. [27]	2, 2, 1	Case 1.–3.: Hydrocephalus	NA	1 month, 1 month, and 1 month	Case 1. Scrotal swelling Case 2: Scrotal swelling, bulging anterior fontanel, and vomiting. Case 3. Scrotal swelling and bulging anterior fontanel.	NA	No
Paterson and Ferch [28]	11	Hydrocephalus and macrocephaly	R	5 weeks	Right scrotal swelling	Abdominal X-ray	No
Nawaz et al. [29]	6	Hydrocephalus secondary to neonatal bacterial meningitis and ventriculitis.	R	4 months	Right scrotal swelling	The US scrotum revealed right-sided hydrocele and VPS end.	No
Bawa et al. [30]	7, 51, 15, 51	Case 1.–4.: Congenital hydrocephalus	R, R, R, L	4, 3, 5, and 4 months	Cases 1.–4.: scrotal swelling	Case 1.–4.: Abdominal X-ray	R, L
Hung et al. [31]	5	Posthemorrhagic hydrocephalus	R	2 months	Right scrotal swelling	Abdominal X-ray	No
Ricci et al. [32]	120	Hydrocephalus	L	72 months	Seven-day history of left scrotal swelling, vomiting, nausea, headache, and fatigue	Abdominal X-ray	L
Shankar et al. [33]	12	Hydrocephalus due to type II Chiari malformation	R	11 months	Right scrotal swelling	Abdominal X-ray	Bilateral
Erikci et al. [34]	48	Hydrocephalus	L	46 months	Left scrotal swelling	Abdominal X-ray	No
Panda et al. [35]	60	Hydrocephalus due to congenital aqueducatal stenosis	L	42 months	Left inguinoscrotal swelling for the last 9 days	Abdominal X-ray	L
Shahizon et al. [36]	168	Congenital hydrocephalus	L	19 months	Left scrotal swelling and fever	Abdominal X-ray and scrotal US	L
Ramareddy et al. [37]	20	Congenital hydrocephalus	L	NA	Scrotal swelling	Abdominal X-ray	No
Gupta et al. [38]	24	Congenital hydrocephalus	R	18 months	Right inguinoscrotal swelling for the last 15 days	Abdominal X-ray	R
Mohammadi et al. [39]	7	Congenital hydrocephalus	R	1 month	Right scrotal swelling	Abdominal X-ray and scrotal US	No
Kita et al. [40]	60	Obstructive (brain tumor) hydrocephalus	L	4 months	Left scrotal swelling	Abdominal X-ray	No
Rahman et al. [41]	48	Hydrocephalus secondary to a pilocytic astrocytoma	R	1 month	Right scrotal swelling	Abdominal X-ray	R
Ward et al. [42]	18	Meningitis resulting in static encephalopathy and hydrocephalus	R	7 months	Right scrotal swelling	Abdominal X-ray and positive transillumination test	No
Öktem et al. [43]	10, 2.5, 9, 2.5	Case 1.–4.: Hydrocephalus	R, R, R, L	4 months, 2.5 months, 4 months, and 1 day	Case 1.–3. Right scrotal swelling Case 4. Left erythematous scrotal swelling	Case 1.–4.: Abdominal x-ray	No
Ammar et al. [44]	6	Hydrocephalus	L	2 months	Scrotal swelling	Abdominal X-ray and positive transillumination test	No
Calvario et al. [45]	24	Hydrocephalus	R	NA	Right scrotal swelling	Abdominal X-ray	No
Kwok et al. [46]	6	Hydrocephalus	L	5 months	Left scrotal swelling	Abdominal X-ray	No
Ram et al. [47]	36	Hydrocephalus secondary to meningitis	R	30 months	Right scrotal swelling	Abdominal X-ray	No
Fuwa et al. [48]	12	Congenital hydrocephalus and holoprosencephaly	L	11 months	Left scrotal swelling	Abdominal X-ray	No
Crofford et al. [49]	9, 3, 5, 48	Case 1.: Hydrocehphalus secondary to subarachnoid hemorrhage Case 2: Hydrocephalus Case 3: Hydrocephalus Case 4.: Hydrocephalus associated with posterior fossa ependymoma	R, R, R, L	8, 2, 1, and 2 months	Case 1. Right scrotal swelling Case 2: Right scrotal swelling Case 3. Right scrotal swelling Case 4. Left scrotal swelling, vomiting, fever, and headache	Case 1.–4.: Abdominal X-ray	No, R, no, no
Bristow et al. [50]	10	Hydrocephalus secondary to aqueductal stenosis	R	1 day	Right scrotal swelling, fever, and pain	Abdominal X-ray, positive transillumination	R
Levey et al. [51]	1	Hydrocephalus secondary to spina bifida and meningomyelocele	R	6 days	Right inguinoscrotal swelling	Abdominal X-ray	No

L = Left; R = Right; US = Ultrasound; CT = Computed tomography; VPS = ventriculoperitoneal shunt.

**Table 6:** Treatment, intraoperative and postoperative complications, length of hospital stay, and mortality of patients with VPS scrotal migration.

Author	Treatment	Intraoperative Complication	Postoperative Complication	Length of Hospital Stay (Days)	Mortality	Follow-Up (Months)
Muhajir et al. [19]	Case 1. Shortening the VPS distal tip catheter and repositioning of VPS into the peritoneal cavity. Case 2: Distal exteriorization of the VPS catheter tip and repositioning of VPS into the peritoneal cavity. Case 3: Shortening the distal tip VPS catheter and repositioning of the VPS into the peritoneal cavity. High ligation of the PV.	None	None	3, 3, 3	0	NA
Javeed et al. [10]	Repositioning of VPS into the peritoneal cavity.	None	None	2	0	3
Topp et al. [20]	Shunt externalization (re-internalized 11 days later), hernia repair, scrotal dehiscence repair, and PV closure.	None	None	3	0	12
Chanchlani et al. [21]	Repositioning of VPS into the peritoneal cavity and hernia repair.	NA	NA	NA	0	NA
Taha et al. [13]	Repositioning of VPS into the peritoneal cavity and PV closure.	None	None	NA	0	6
Alkhudari et al. [3]	Spontaneous resolution of the VPS without intervention at the day of admission.	Not applicable	Not applicable	1	0	1
Ahmed et al. [22]	Repositioning of VPS into the peritoneal cavity.	None	None	2	0	2
Hauser et al. [23]	Manual (non-operative) reposition of shunt catheter and hernia repair.	None	None	1	0	NA
Abdoli et al. [24]	All cases: Repositioning of VPS into the peritoneal cavity and hernia repair.	None	None	2, 2, 3, NA	0	NA
Agarwal et al. [25]	Repositioning of VPS into the peritoneal cavity.	None	NA	NA	0	NA
Dharmajaya [26]	Repositioning of VPS into the peritoneal cavity and PV closure.	None	None	7	0	NA
Ezzat et al. [27]	Repositioning of VPS into the peritoneal cavity (1 laparotomy, and 2 laparoscopy).	None	None	NA	0	NA
Paterson et al. [28]	Repositioning of VPS into the peritoneal cavity. One week later, due to a recurrence of catheter migration in the right scrotum, a revision operation was made in which the distal end of the catheter was shortened.	None	None	NA	0	NA
Nawaz et al. [29]	Bilateral herniotomy, left-sided orchidopexy, and repositioning of the VPS tip into the peritoneal cavity.	None	None	NA	0	4
Bawa et al. [30]	Case 1.–4.: Repositioning of VPS into the peritoneal cavity and hernia repair.	None	NA	NA	0	NA
Hung et al. [31]	Repositioning of VPS into the peritoneal cavity and PV closure.	None	None	4	0	NA
Ricci et al. [32]	Repositioning of VPS into the peritoneal cavity and hernia repair.	None	None	NA	0	NA
Shankar et al. [33]	Repositioning of VPS into the peritoneal cavity and hernia repair.	None	None	NA	0	NA
Erikci et al. [34]	Repositioning of VPS into the peritoneal cavity.	None	None	NA	0	120
Panda et al. [35]	Repositioning of VPS into the peritoneal cavity and hernia repair.	None	None	2	0	24
Shahizon et al. [36]	Repositioning of VPS into the peritoneal cavity and hernia repair.	None	None	NA	0	NA
Ramareddy et al. [37]	Spontaneous resolution of the VPS without intervention.	Not applicable	Not applicable	NA	0	36
Gupta et al. [38]	Repositioning of VPS into the peritoneal cavity and hernia repair.	None	None	NA	0	NA
Mohammadi et al. [39]	Repositioning of the shunt into the peritoneal cavity.	None	None	NA	0	6
Kita et al. [40]	Manual (non-operative) reposition of shunt catheter. Prophylactic obliteration of the PV.	NA	NA	NA	0	NA
Rahman et al. [41]	Repositioning of VPS into the peritoneal cavity and hernia repair.	None	None	NA	0	NA
Ward et al. [42]	Repositioning of VPS into the peritoneal cavity. The PV was doubly clamped, transected, and highly ligated.	None	None	NA	0	NA
Öktem et al. [43]	Case 1.–4.: Repositioning of VPS into the peritoneal cavity, and PV closure.	None	None	NA	0	NA
Ammar et al. [44]	VPS removal and antibiotic therapy. Insertion of VPS a few weeks later.	None	None	NA	0	12
Calvario et al. [45]	Repositioning of the shunt into the peritoneal cavity.	None	None	NA	0	NA
Kwok et al. [46]	Repositioning of VPS into the peritoneal cavity and PV closure.	None	None	NA	0	NA
Ram et al. [47]	Repositioning of the shunt into the peritoneal cavity.	None	None	NA	0	NA
Fuwa et al. [48]	Repositioning of the new shunt into the peritoneal cavity.	None	None	10	0	NA
Crofford et al. [49]	Case 1. Repositioning of the shunt into the peritoneal cavity Case 2: Repositioning of VPS into the peritoneal cavity and hernia repair. Case 3: Repositioning of the shunt into the peritoneal cavity Case 4: Spontaneous resolution of the VPS without intervention	None	None	NA	NA	NA
Bristow et al. [50]	Shortening the distal tip catheter and repositioning of shunt into the peritoneal cavity. (Hernia repair 3 months later).	None	None	NA	0	3
Levey et al. [51]	Repositioning of the shunt into the peritoneal cavity and PV closure.	None	None	NA	0	NA

NA = Not available; PV = Processus vaginalis; VPS = ventriculoperitoneal shunt.

## Data Availability

The data supporting the findings of the literature review are available from the corresponding author upon reasonable request, in accordance with ethical standards and data privacy regulations.
